# Imported Dengue Virus Serotype 3, Yemen to Italy, 2010

**DOI:** 10.3201/eid1705.101626

**Published:** 2011-05

**Authors:** Paolo Ravanini, Eili Huhtamo, Essi Hasu, Felicita Rosa, Stefano Costantino, Maria G. Crobu, Valentina Ilaria, Anna M. Nicosia, Pietro L. Garavelli, Olli Vapalahti

**Affiliations:** Author affiliations: Azienda Ospedaliero-Universitaria Maggiore della Carità, Novara, Italy (P. Ravanini, F. Rosa, S. Costantino, M.G. Crobu, V. Ilaria, A.M. Nicosia, P.L. Garavelli);; University of Helsinki, Helsinki, Finland (E. Huhtamo, E. Hasu, O. Vapalahti)

**Keywords:** dengue virus, serotype 3, Yemen, Italy, traveler, viruses, letter

**To the Editor:** Dengue is a mosquito-transmitted viral disease endemic to the tropics and subtropics worldwide. It is caused by 4 dengue virus serotypes (DENV-1–4) that belong to the genus *Flavivirus*. The disease varies from dengue fever to life-threatening hemorrhagic fever and shock that are associated with secondary infections. During recent decades, dengue incidence and prevalence have increased in disease-endemic areas, and the disease has been increasingly recognized in travelers (1). We report a case of dengue in a man who had traveled to Yemen.

In June 2010, a 38-year-old Italian man was admitted to the hospital for high fever (maximum 39.5°C) after a 1-week work-related stay in Yemen, near Mukalla, in the province of Hadhramaut. The patient had visited the countryside where he was heavily bitten by mosquitoes.

On the third day after onset of fever, the patient started to experience strong and unremitting frontal and retro-orbital headache and joint pains, which lasted for 5 days. He also experienced vomiting. Laboratory test results showed mild leukopenia (2.41 × 10^3^ cells/mm^3^) and lowered platelet counts (96 × 10^3^ cells/mm^3^), increased liver alanine aminotransferase levels (151 U/L), and mildly abnormal blood clotting (prothrombin time, international normalized ratio 1.24). In 1 week, the patient started to recover and was discharged from the hospital. The patient received antimicrobial (levoxacin) and antipyretic (acetaminophen) drugs. Laboratory testing after discharge showed increased levels of hepatic enzymes, which reached maximum levels on day 13 after onset of symptoms (alanine aminotransferase 669 U/L) and decreased to within reference limits in 1 month.

A plasma sample taken on day 6 after disease onset was positive for flavivirus RNA by reverse transcription–PCR (RT-PCR) specific for members of the genus *Flavivirus* (2). The RT-PCR product was sequenced, and according to BLAST (www.ncbi.nlm.nih.gov/blast), the 184-bp sequence obtained shared 99% nt identity with dengue serotype 3 viruses in GenBank. The plasma sample also had positive results for dengue virus nonstructural protein 1 (NS1) antigen test (Platelia NS1 Ag ELISA; Bio-Rad, Marnes-la-Coquette, France), anti-dengue immunoglobulin (Ig) M ELISA (Focus Technologies, Cypress, CA, USA), and in an in-house IgG immunofluorescence assay that used DENV-3–infected Vero E6 cells as antigens (titer 40). Other concomitant infections were ruled out by bacterial cultures and by laboratory tests for various viral, bacterial, and parasitic pathogens.

Virus isolation was conducted as described (3). Viral RNA was extracted from the supernatant of the infected Vero E6 cells, and the envelope gene was amplified in an RT-PCR. The amplified product was directly sequenced (details available from P.R. upon request). The obtained envelope gene sequence (GenBank accession no. HQ336219) of 1,479 bp was aligned with 26 other DENV-3 strains, including the most similar sequences identified in nucleotide BLAST search and a global set of sequences representing different genotypes (4), by using MUSCLE (www.ebi.ac.uk/Tools/muscle/index.html). A neighbor-joining phylogenetic tree was inferred by using p-distance, with 1,000 bootstrap replicates in MEGA version 4 (www.megasoftware.net).

The strain isolated from Yemen in 2010 (this study) shared highest nucleotide homologies with strains from Jeddah, Saudi Arabia (98%–99%), and Tanzania (98%) and was phylogenetically grouped within genotype III of DENV-3. The most closely related strains also included recent isolates from Côte d’Ivoire, People’s Republic of China, Bhutan, and India (Figure).

**Figure:**
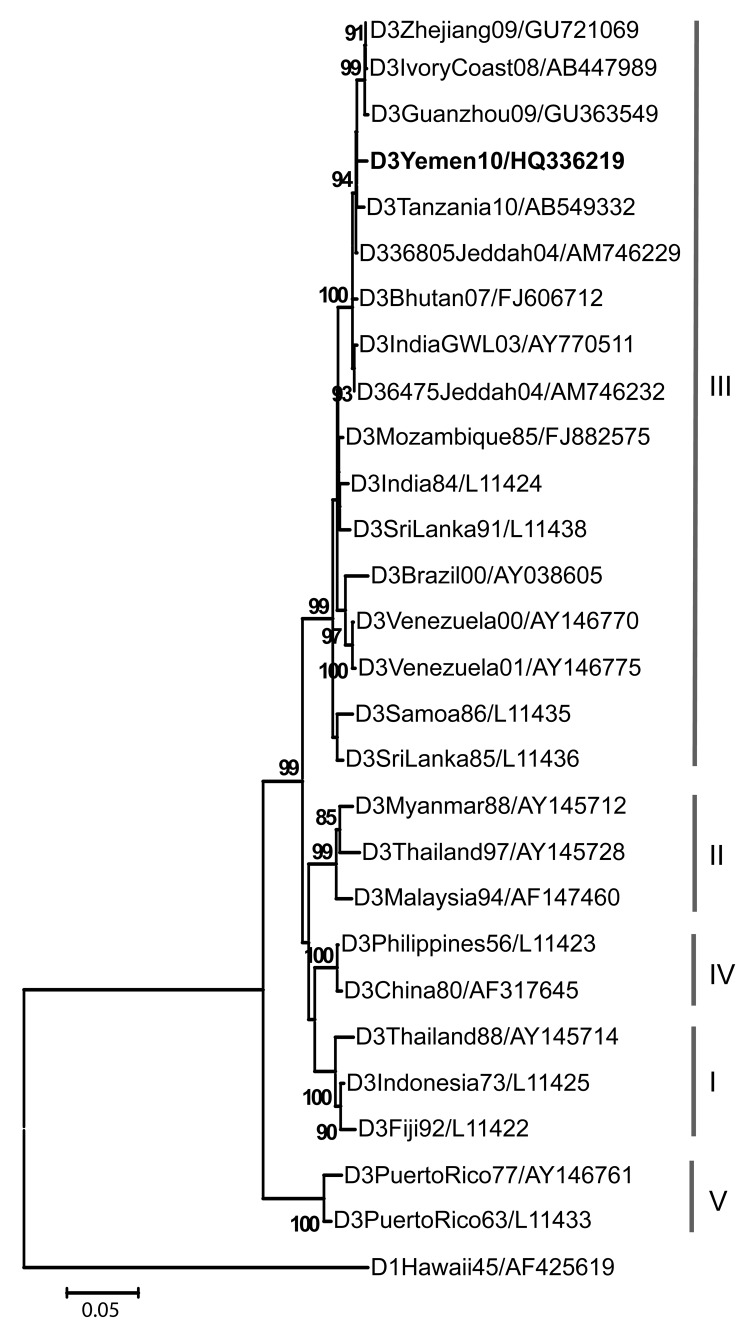
Neighbor-joining phylogenetic tree based on complete envelope gene sequences of dengue virus (DENV) serotype 3 virus, rooted with DENV-1. Bootstrap support values >80 are shown. **Boldface i**ndicates the 2010 isolate from Yemen. Scale bar represents nucleotide substitutions per site. Virus abbreviations are dengue virus type/origin/year/GenBank accession number.

Dengue has been documented in the Middle East, including Saudi Arabia (5) and Yemen (6). In May 2010, a dengue outbreak in Yemen was reported (7). The patient reported here had visited Yemen in June, and by August the outbreak had resulted in ≈100,000 infections and 200 deaths (7). To our knowledge, the DENV strains involved in this outbreak had not been previously characterized. The same genotype as the isolate described here, genotype III of DENV-3, was most recently isolated from Saudi Arabia in 2005 and has been associated with recent outbreaks in Sri Lanka, East Africa, and Latin America (8). Without further information, it remains unknown whether other serotypes or genotypes circulate concurrently in Yemen.

According to the Italian Public Health Institute (Istituto Superiore di Sanitá), the numbers of imported dengue cases in Italy are increasing (www.iss.it/binary/publ/cont/09_11web.pdf), but because only a few hospitals perform diagnostic tests, dengue is likely to be underdiagnosed in Italy. Viremic travelers can contribute to spread of DENV, and during the active mosquito season, travelers from dengue-endemic areas who have diagnosed or suspected dengue should be advised to avoid contact with mosquitoes.

Recently, indigenous transmission of dengue virus was shown to have occurred in Côte d’Azur in southern France (9) and in Croatia (10), thereby highlighting the risk in areas that have *Aedes albopictus* mosquitoes, which are competent DENV vectors. In these areas, including Italy, vector control and surveillance of DENV in mosquitoes should be conducted. We conclude that recognition and diagnosis of dengue in travelers should be emphasized and that characterization of DENV strains from travelers helps elucidate the molecular epidemiology of DENV in a global context.
